# Adding Hyponatremia to the “Rule-of-6” Prediction Tool Improves Performance in Identifying Hospitalised Patients with COVID-19 at Risk of Adverse Clinical Outcomes

**DOI:** 10.3390/pathogens13080694

**Published:** 2024-08-16

**Authors:** Meng Ying Sim, Jinghao Nicholas Ngiam, Matthew Chung Yi Koh, Wilson Goh, Srishti Chhabra, Nicholas W. S. Chew, Louis Yi Ann Chai, Paul Anantharajah Tambyah, Ching-Hui Sia

**Affiliations:** 1 Division of Infectious Diseases, Department of Medicine, National University Hospital, National University Health System, Singapore 119228, Singapore; mengying.sim@mohh.com.sg (M.Y.S.); nicholas_ngiam@nuhs.edu.sg (J.N.N.); matthew.koh@mohh.com.sg (M.C.Y.K.); wilson.goh@mohh.com.sg (W.G.); srishti.chhabra@mohh.com.sg (S.C.); louis_chai@nuhs.edu.sg (L.Y.A.C.); mdcpat@nus.edu.sg (P.A.T.); 2 Department of Cardiology, National University Heart Centre Singapore, Singapore 119074, Singapore; nicholas_ws_chew@nuhs.edu.sg; 3 Department of Medicine, Yong Loo Lin School of Medicine, National University of Singapore, Singapore 117597, Singapore; 4 Infectious Diseases Translational Research Programme, Department of Medicine, Yong Loo Lin School of Medicine, National University of Singapore, Singapore 117597, Singapore

**Keywords:** COVID-19, hyponatremia, predict, outcomes, Singapore

## Abstract

The ‘rule-of-6’ prediction tool was shown to be able to identify COVID-19 patients at risk of adverse outcomes. During the pandemic, we frequently observed hyponatremia at presentation. We sought to evaluate if adding hyponatremia at presentation could improve the ‘rule-of-6’ prediction tool. We retrospectively analysed 1781 consecutive patients admitted to a single tertiary academic institution in Singapore with COVID-19 infection from February 2020 to October 2021. A total of 161 (9.0%) patients had hyponatremia. These patients were significantly older, with more co-morbidities and more likely to be admitted during the Delta wave (2021). They were more likely to have radiographic evidence of pneumonia (46.0% versus 13.0%, *p* < 0.001) and more adverse outcomes (25.5% vs. 4.1%, *p* < 0.001). Hyponatremia remained independently associated with adverse outcomes after adjusting for age, lack of medical co-morbidities, vaccination status, year of admission, CRP, LDH, and ferritin. The optimised cut-off for serum sodium in predicting adverse outcomes was approximately <135 mmol/L as determined by the Youden index. Although derived in early 2020, the ‘rule-of-6’ prediction tool continued to perform well in our later cohort (AUC: 0.72, 95%CI: 0.66–0.78). Adding hyponatremia to the ‘rule-of-6’ improved its performance (AUC: 0.76, 95%CI: 0.71–0.82). Patients with hyponatremia at presentation for COVID-19 had poorer outcomes even as new variants emerged.

## 1. Introduction

Since the onset of the coronavirus disease 2019 (COVID-19) pandemic, several clinical risk scores have been developed to predict adverse outcomes in COVID-19. Existing scores for community-acquired pneumonia such as Pneumonia Severity Index and CURB-65 also showed some efficacy in predicting mortality and need for intensive care in patients with COVID-19 pneumonia [1], while novel clinical risk scores were developed specifically for COVID-19 in order to improve the prognostication of these patients. The “rule-of-6” tool, which is one such example of a novel risk score, was first adopted by Dickens et al. (2021) in a tertiary academic medical centre in Singapore between February and April 2020 when the wild-type “Wuhan” strain was dominant, to identify patients at risk of COVID-19 disease progression [2]. The ‘rule-of-6’ prediction tool, which consists of three main components (C-reactive protein, CRP > 60 mg/L; lactate dehydrogenase, LDH > 600 U/L; and ferritin > 600 mcg/L), was shown to be able to identify patients at risk of adverse outcomes with high area under receiver operating characteristic curves (AUC, ROCs) at 0.99, 0.88, and 0.90, respectively.

Later in the pandemic, as different variants of SARS-CoV-2 predominated, we observed the increasing prevalence of hyponatremia (low serum sodium) at presentation. This may be due to multiple mechanisms, with syndromes of inappropriate antidiuretic hormones possibly contributing up to 50% of cases [3] and volume depletion or hyperinflammatory states leading to multi-organ dysfunction being the other main aetiologies [4]. Recent studies including meta-analyses have found that hyponatremia was associated with poor outcomes in patients with COVID-19 [5,6]. This raised the potential for including hyponatremia as a component into existing clinical risk scores to improve their performance in a more contemporary cohort.

As the pandemic progressed, Singapore had built and implemented purpose-built isolation facilities to care for patients with COVID-19 who were medically stable and did not require a tertiary level of hospital care [7]. For example, some of these patients required a short course of rehabilitation due to functional decline, while others required the administration of intravenous remdesivir, which could not be performed in the outpatient setting at certain periods of time. These facilities helped reduce the impact on hospitals and were a regular feature in the nation’s approach towards managing COVID-19. Patients who were well enough to recuperate at home were also discharged directly from emergency departments. Risk stratification served an important purpose in helping the clinician and hospital triage the level of medical care required for patients with COVID-19. Such tools may be useful during large surges in case numbers where hospital resources are stretched to the limit. Therefore, we sought to evaluate if adding hyponatremia at presentation could improve performance of the previously developed ‘rule-of-6’ prediction tool.

## 2. Materials and Methods

We retrospectively analysed 1781 consecutive patients with polymerase chain reaction-positive COVID-19 infection hospitalised for SARS-CoV-2 from February 2020 to October 2021. We excluded patients seen in the emergency department who did not require hospital admission or were directly transferred to community care isolation facilities. Basic demographic factors, clinical presentation, and laboratory results (such as serum sodium concentration, full blood count, C-reactive protein, etc.) obtained within 24 h of admission for each patient and clinical outcomes were obtained. We then followed up on their individual progress and eventual outcomes including the need for COVID-19 therapeutics, oxygen supplementation, intensive care, acute kidney injury, and mortality. Adverse outcomes were defined as requiring supplemental oxygenation, intensive care, or mortality.

An optimised cut-off for serum sodium in identifying adverse clinical outcomes (requiring oxygenation, intensive care, or mortality) was also derived by the Youden index. We then divided the study population based on those with or without hyponatremia at the appropriate cut-off. We subsequently analysed differences between the two groups. A multivariable model was constructed to identify independent predictors of adverse outcomes. We adjusted for the following confounders (older age, lack of medical co-morbidities, COVID-19 vaccination, year of admission, elevated CRP, ferritin, LDH, and hyponatremia) as most of them had previously been shown to be important predictors of adverse outcomes in patients with COVID-19 [8,9,10]. We constructed three receiver operating characteristic curves (ROCs) to compare the performance of various parameters in predicting adverse outcomes by comparing the area under the curve (AUC): (1) hyponatremia alone; (2) “rule-of-six” prediction tool; and (3) “rule-of-six” prediction tool + hyponatremia.

T-tests were used for continuous variables and chi-squared tests were used for categorical variables to compare differences in characteristics between the different groups. Data for continuous variables were expressed as means (±1 standard deviation), while data for categorical variables were expressed as frequencies (and percentages). A *p*-value of less than 0.05 was considered significant in this study. Data analyses were performed with SPSS version 20.0 (SPSS, Inc., Chicago, IL, USA). Approval was sought from the National Healthcare Group Domain Specific Review Board (DSRB 2020/00545) and our study was conducted in line with the principles laid out by the Declaration of Helsinki. All data were anonymised, and a waiver of written informed consent was obtained prior to the conduct of this study.

## 3. Results

A total of 161 (9.0%) patients had hyponatremia. These patients were significantly older (51.9 ± 16.9 vs. 41.2 ± 14.0 years, *p* < 0.001) but were similar in terms of the male gender proportion (77.4% vs. 80.5%, *p* = 0.338). These patients were more likely to have at least one medical co-morbidity (51.6% vs. 18.5%, *p* < 0.001) of which hypertension was most common (33.5% vs. 12.3%, *p* < 0.001), followed by hyperlipidaemia (25.5% vs. 8.1%, *p* < 0.001) and diabetes (28.8% vs. 5.2%, *p* < 0.001). They also had higher C-reactive protein (43.6 ± 62.1 vs. 11.9 ± 22.0 mg/L, *p* < 0.001), ferritin (456.4 ± 513.7 vs. 183.9 ± 249.7 mcg/L, *p* < 0.001), and lactate dehydrogenase (513.3 ± 242.0 vs. 410.5 ± 262.4 U/L, <0.001).

A larger proportion of patients who had hyponatremia were more likely to have been admitted during the Delta wave in 2021 (36.0% vs. 22.4%, *p* < 0.001). They were more likely to have radiographic evidence of pneumonia (46.0% versus 13.0%, *p* < 0.001), more acute kidney injury (9.9% versus 3.6%, *p* < 0.001), and more adverse outcomes including requiring oxygenation, intensive care, or mortality (25.5% vs. 4.1%, *p* < 0.001). Proportions of vaccinated patients did not differ significantly between the groups (19.9% vs. 14.9%, *p* = 0.093). Patients with hyponatremia were also more likely to receive COVID-19 therapeutics such as remdesivir (20.5% vs. 3.3%, *p* < 0.001) and dexamethasone (14.3% vs. 2.1%, *p* < 0.001). There were no significant differences in baricitinib (1.2% vs. 0.4%, *p* = 0.192) and tocilizumab (0.0% vs. 0.2%, *p* = 0.999) received but this was likely due to the small number of patients who received them (Table 1). The optimised cut-off for serum sodium in predicting adverse outcomes was approximately <135 mmol/L, as determined by the Youden index.

**Figure 1:**
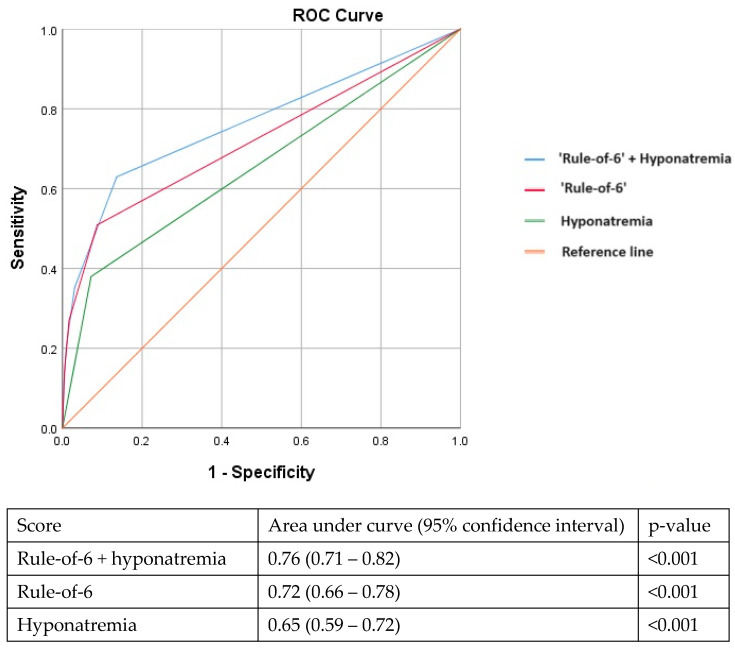
Adding hyponatremia to the ‘rule-of-6’ prediction tool improves the area under the receiver operating characteristic curve in identifying patients at risk for adverse outcomes.

In the multivariable analysis, hyponatremia (serum Na < 135 mmol/L) at presentation remained independently associated with adverse outcomes after adjusting for older age, vaccination status, lack of medical co-morbidities, year of admission, elevated CRP, LDH, and ferritin levels (Table 2). Although derived in early 2020, the ‘rule-of-6’ prediction tool continued to perform well in our later cohort (AUC: 0.72, 95%CI: 0.66–0.78). Adding hyponatremia to the ‘rule-of-6’ improved its performance further with an incremental increase in the AUC (AUC: 0.76, 95%CI: 0.71–0.82) (Figure 1).

## 4. Discussion

In this retrospective analysis, we found several key findings: (1) Hyponatremia was associated with poorer clinical outcomes. (2) Patients infected by COVID-19 in 2021 were more likely to have hyponatremia, compared with patients infected in 2020. (3) Adding hyponatremia to the ‘rule-of-6’ improved its performance in predicting patients who would experience adverse clinical outcomes in the later waves of COVID-19 infection.

Various systematic reviews have looked at the association between hyponatremia and COVID-19. A systematic review by Khidir et al., 2022, found that the prevalence of hyponatremia in COVID-19 patients was 25.8%, and it was significantly associated with increased odds for mortality, ICU admission, ventilation, and length of stay [4]. In another meta-analysis by Akbar et al., 2021, hyponatremia incidence was 24% and was found to be associated with poor outcomes [5]. In this study, we demonstrated that hyponatremia had additional predictive value on top of an existing risk score for COVID-19. This may lead to potential clinical application in prognostication.

Hyponatremia is one of the most common electrolyte disorders and has been shown to be associated with increased morbidity and mortality. Even a moderate decrease in serum sodium is associated with an increased risk of mortality [6]. Hyponatremia exists in three different states—hypovolemic, euvolemic, and hypervolemic states—with largely different pathophysiology. The antidiarrheic hormone and renin–angiotensin–aldosterone system work in harmony, which affects the sodium and water balance in our body. The syndrome of an inappropriate secretion of the antidiuretic hormone is a possible explanation for the development of hyponatremia [11]. Hyperinflammatory states have also been postulated to cause nonsuppressible vasopressin secretion even in the hypotonic state. This is mainly driven by a high interleukin-6 level, which impairs the osmoregulatory mechanism of ADH in the brain, causing hyponatremia [12]. Our findings are consistent with this hypothesis, where patients with lower serum sodium were more likely to have higher serum CRP, ferritin, and LDH levels. Kidney injury as a result of COVID-19 can also result in hyponatremia due to proximal convoluted tubule malfunction, either from hemodynamic changes or from direct viral effects [13]. Our study showed an association between hyponatremia and acute kidney injury.

Multiple studies have been performed to identify predictors of poor clinical outcomes. Age is a well-known risk factor for severe COVID-19 outcomes [14]. Other factors such as inflammatory markers have also been well studied and in an earlier study, a hyperinflammatory phenotype (CRP > 17 mg/L) was independently associated with adverse clinical outcomes [8]. A number of studies have focused on simple and cost-effective means of risk stratification and triage, for example, using the creatinine–lymphocyte ratio in predicting outcomes in hospitalised patients with COVID-19 [15]. With these principles in mind, our study sought to include both inflammatory markers and relatively low-cost tools such as serum sodium levels to derive a useful clinical risk prediction tool that can be readily applied to most patients with COVID-19 infection.

There could be multiple reasons as to why hyponatremia was more common in 2021. Firstly, this could be due to the changing demographics as well as admission policies. As the COVID-19 pandemic progressed, our understanding of the virus continued to evolve, which was reflected in management of patients. In Singapore, the differing demographics and characteristics of the epidemic changed significantly (initially constituting young migrant workers, which subsequently shifted to the more vulnerable, elderly population within the local community) [9]. The elderly may potentially be more prone to kidney injury, and severe disease (and hence hyperinflammatory states). Also in 2020, Singapore hospitalised almost all patients diagnosed with COVID-19 including those who were asymptomatic; this changed in late 2020 and 2021 when those who did not need hospitalisation or were at low risk of complications were sent to community isolation facilities. Second, with the Delta strain being more prevalent in 2021, there could have been differing effects of the virus on the osmotic regulation and kidney injury.

In a young population with few co-morbidities, adding the presence of fever as a parameter to the CHA2DS2-VASc or the ISARIC 4C prognostic score improved the performance in predicting more severe COVID-19 disease [10]. Similarly, in this study population with relatively mild disease (only 4.8% required oxygen supplementation and 3.1% required intensive care), adding hyponatremia to the ‘rule-of-6’ prediction tool improved its performance in identifying patients at risk for adverse outcomes.

There were several limitations of this study. This was a single-centre study involving only hospitalised patients in our institution. A vast majority of our patients in 2020 were young and fit migrant workers, who were compared against a more vulnerable and elderly local population in 2021. Therefore, the study population was heterogenous. As a result, we were not able to study the prevalence of hyponatremia and its association with outcomes of patients with very mild illness especially in 2021 as most of those patients were not hospitalised. Over the years of this study, changes in the predominant circulating strain as well as the introduction of effective vaccination against COVID-19 would likely also have an effect on clinical outcomes. Although we adjusted for the vaccination status, we could not adjust for the differences in viral strains. The patients included in this study were hospitalised prior to October 2021, before the Omicron variant became dominant. Therefore, the trends observed in this study may not be conclusively extrapolated to the more contemporary COVID-19 variants. We were unable to include all medical co-morbidities as separate covariates in our multivariable model as we were limited by sample size and it would have led to overfitting of the model. Nevertheless, we were able to adjust for the lack of medical co-morbidities, as well as other important covariates that had been previously demonstrated to predict adverse outcomes in COVID-19 based on prior work. In the multivariable model, we showed that hyponatremia remained independently associated with adverse outcomes [8,9,10]. We did not compare this risk score with other existing clinical scores, as this had not been the focus of our current study. Future prospective research would be important to explore the clinical utility of various risk scores in the prognostication of patients presenting with COVID-19. Furthermore, our findings remain exploratory. This is a derivation cohort that proposes that hyponatremia in addition to the rule-of-six can provide better prognostication for patients with COVID-19. Future prospective studies with a validation cohort would be important to verify our findings.

## 5. Conclusions

Hyponatremia was observed amongst patients with COVID-19, particularly with the Delta variant of SARS-CoV-2. Patients with hyponatremia at presentation had significantly poorer clinical outcomes. Adding hyponatremia as a parameter to the ‘rule-of-6’ prediction tool improved its prognostic performance. Future prospective research is needed to evaluate if this applies to current circulating SARS-CoV-2 variants.

## Figures and Tables

**Table 1:** Characteristics of hospitalised patients with COVID-19, with or without hyponatremia at presentation (serum sodium < 135 mmol/L).

Parameter	Hyponatraemia (n = 161)	No Hyponatraemia (n = 1620)	*p*-Value
*Baseline characteristics*			
Age (years)	51.9 (±16.9)	41.2 (±14.0)	<0.001
Admitted in 2021 (compared with admission in 2020)	58 (36.0%)	363 (22.4%)	<0.001
Cycle threshold value of initial nasopharyngeal swab polymerase chain reaction for SARS-CoV-2	20.5 (±6.6)	22.0 (±14.4)	0.479
Absolute lymphocyte count (×10^9^/L)	1.5 (±0.9)	1.9 (±1.4)	<0.001
C-reactive protein (mg/L)	43.6 (±62.1)	11.9 (±22.0)	<0.001
Ferritin (mcg/L)	456.4 (±513.7)	183.9 (±249.7)	<0.001
Lactate dehydrogenase (U/L)	513.3 (±242.0)	410.5 (±262.4)	<0.001
Serum sodium (mmol/L)	131.9 (±2.6)	138.5 (±1.9)	<0.001
Serum potassium (mmol/L)	3.8 (±0.4)	4.0 (±3.6)	0.443
Serum urea (mmol/L)	4.9 (±3.5)	3.9 (±3.0)	<0.001
Serum creatinine (mmol/L)	83.3 (±49.6)	79.0 (±53.3)	0.333
HbA1c (%)	7.8 (±2.6)	5.9 (±1.3)	<0.001
Sex (male)	123 (77.4%)	1303 (80.5%)	0.338
Smoker	17 (11.0%)	113 (7.3%)	0.104
Hypertension	54 (33.5%)	200 (12.3%)	<0.001
Hyperlipidaemia	41 (25.5%)	132 (8.1%)	<0.001
Diabetes mellitus	45 (28.8%)	85 (5.2%)	<0.001
Ischaemic heart disease	13 (8.1%)	38 (2.3%)	<0.001
No past medical history	78 (48.4%)	1321 (81.5%)	<0.001
Vaccinated against COVID-19	32 (19.9%)	241 (14.9%)	0.093
Asymptomatic	14 (8.7%)	293 (18.1%)	0.003
Persistent fever beyond 72h	48 (29.8%)	156 (9.6%)	<0.001
Presence of pneumonia	74 (46.0%)	211 (13.0%)	<0.001
*COVID-19 therapeutics*			
Remdesivir	33 (20.5%)	53 (3.3%)	<0.001
Dexamethasone	23 (14.3%)	23 (2.1%)	<0.001
Baricitinib	2 (1.2%)	7 (0.4%)	0.192
Tocilizumab	0 (0.0%)	3 (0.2%)	0.999
Received COVID-19 therapeutics	37 (23.0%)	61 (3.8%)	<0.001
*Clinical outcomes*			
Length of hospital stay (days)	2.2 (±2.5)	1.0 (±2.0)	<0.001
Requiring supplemental oxygenation	36 (22.4%)	50 (3.1%)	<0.001
Acute kidney injury	16 (9.9%)	59 (3.6%)	<0.001
Required intensive care or experienced mortality	27 (16.8%)	33 (2.0%)	<0.001
Mortality	4 (2.5%)	9 (0.6%)	0.024
Composite adverse outcome (requiring supplemental oxygenation, intensive care or mortality)	41 (25.5%)	67 (4.1%)	<0.001

**Table 2:** Multivariable model showing clinical and laboratory parameters associated with composite adverse outcomes.

Parameter	Adjusted Odds Ratio (95% Confidence Interval)	*p*-Value
Older age (per year)	1.03 (1.01–1.05)	0.003
No past medical history	0.29 (0.16–0.54)	<0.001
Received COVID-19 vaccination	0.81 (0.34–1.95)	0.639
Hospital admission in 2021 (compared with 2020)	1.42 (0.63–3.24)	0.399
Elevated C-reactive protein (mg/L)	1.01 (1.01–1.02)	<0.001
Elevated ferritin (mcg/L)	1.00 (1.00–1.01)	0.245
Elevated lactate dehydrogenase (U/L)	1.00 (1.00–1.01)	0.012
Hyponatremia (serum Na < 135 mmol/L) at presentation	2.60 (1.45–4.67)	0.001

## Data Availability

The datasets generated during and/or analysed during the current study are not publicly available, but are available from the corresponding author on reasonable request. Data are currently processed in a format unsuitable to be uploaded into an online repository. However, the data may be made available on reasonable request to the corresponding author.
